# Quality of life in a family-centric applied behavior analysis model: A case series study

**DOI:** 10.1371/journal.pone.0329939

**Published:** 2025-08-06

**Authors:** Madalina Ciobanu, Gina Barnes, Natalie J. Castell, Robert P. Adelson, Anurag Garikipati, Navan Preet Singh, Qingqing Mao, Ritankar Das

**Affiliations:** Research and Development, Montera, Inc. dba Forta, San Francisco, California, United States of America; Marwadi University, INDIA

## Abstract

Autism spectrum disorder (ASD) is a condition with growing prevalence that results in significant healthcare spending, reduced parent income, high levels of family stress, and decreased quality of life (QoL). QoL is a measure for assessing overall wellness, and in the context of ASD is related to parent self-efficacy. Validated treatments, of which applied behavior analysis (ABA) is the gold standard, can mitigate some of the consequences that may be detrimental to parental QoL. We examined changes in parental QoL across multiple domains within three parent-child dyads in the context of a parent-led ABA model where the treatment was delivered by the parent. We hypothesized that parental QoL would be increased concurrently with ABA treatment lowering the frequency of interfering behaviors, which contribute to parental stress and limited self-efficacy. QoL components improved between the first and follow-up evaluations, though not all QoL component score changes achieved clinical significance. This study demonstrates a correlation between parental QoL and clinical treatment progress; with further validation, QoL assessment may serve as a tool to guide individualized treatment approaches that improve family outcomes.

## Introduction

Autism spectrum disorder (ASD) is a neurodevelopmental condition identified in a growing number of children in the US, affecting 1 in 36 children 8 years of age [1,2]. ASD is characterized primarily by struggles in communicating, restricted, repetitive patterns of behavior, and heightened responses to sensory stimuli [3]. The consequences of ASD are lifelong and pervasive, occurring at societal, family, and individual levels [4–8]. These consequences include the significant economic burden to the healthcare system and parents or caregivers (hereafter referred to as parents), resulting from direct and indirect costs (e.g., lost wages) [9], as well as increased stress and decreased quality of life (QoL) for parents associated with the allostatic load of caring for a child on the autism spectrum [3].

QoL encompasses overlapping domains that span financial, emotional, social, and physical well-being [10]. QoL is a broadly important construct, as it can indicate the effectiveness of a health intervention, and can thereby guide the intervention’s design to maximize patient and family outcomes [11,12]. In the context of ASD, previous research investigating stress and QoL in families with children on the autism spectrum has demonstrated the interrelatedness of QoL, parental self-efficacy, and ASD symptom severity [13,14]. In particular, the severity of ASD symptoms and specific interfering behaviors (IBs) have been associated with decreased parent self-efficacy and QoL [13,15,16]. The Child and Family Quality of Life-Second Edition (CFQL-2) scale is a validated assessment tool to assess QoL, and was developed specifically for use in the context of ASD and other neurodevelopmental conditions [15]. Its intended use includes tracking the outcomes of interventions targeted at improving family QoL. By assessing specific domains that contribute to QoL, it has been utilized to understand the interdependent relationship between contributing factors. For example, how challenges that induce stress diminish the ability of family members to respond productively to the needs of their child on the autism spectrum, which, in turn, can result in escalation of IBs [17].

Validated treatment approaches, such as applied behavior analysis (ABA), help to decrease the incidence of IBs (e.g., elopement) and increase the use of adaptive behaviors (e.g., communicating needs) in children on the autism spectrum [18]. ABA accomplishes this through identifying the underlying purpose of IBs and teaching children effective coping strategies to replace them [18]. Intuitively, ABA can also serve as a tool to improve family QoL by reducing the frequency of IBs that contribute to parental stress.

One challenge parents face that contributes to stress and decreased self-efficacy is a lack of knowledge and training regarding appropriate treatment for ASD [19,20]. One possible solution is to train the parents to become qualified behavior technicians (BTs) to deliver treatment for their own child, under a parent behavior technician (pBT) model of care [21–28]. Our company (an ABA treatment provider) has adopted the pBT model as an option for parents that face significant barriers (e.g., geographical, financial, etc.) in obtaining appropriate treatment for their child. The pBT model of treatment delivery is intended to empower parents and increase parental self-efficacy by providing parents with the necessary expertise to apply ABA methods within the standard of care (SOC). This enables them to effectively communicate with and engage in naturalistic teaching for behavior changes with their child on the autism spectrum. In this case series, we report on changes in CFQL-2 for three parent-child dyads after >4.5 months of treatment using the pBT model in association with data on individual patient treatment progress. We hypothesized that the parents of children receiving treatment under the pBT model would experience improved QoL correlated with the clinical progress of their child and, in particular, with reduced incidence of IBs. This preliminary case series suggests that a larger-scale investigation of the ability of the pBT model to improve family QoL is warranted.

## Methods

### Study design

This longitudinal case series utilizes QoL data collected from parents of incoming patients prior to treatment initiation, and subsequently after the parent has become a pBT and administered treatment to their child (the patient). Patient data was collected observationally during the course of pBT-led treatment. Self-reported pBT data included demographic information (race/ethnicity, sex assigned at birth); patient data included demographic information (age, race/ethnicity, sex assigned at birth), insurance type (public, private), ASD severity level classification (mild, moderate, severe), prescribed ABA treatment hours (i.e., treatment plan type), delivered weekly treatment hours (i.e., treatment utilization), and the clinical progress of the patient. Family demographics and patient ASD severity data were collected as part of the patient intake process. Our proprietary dataset of treatment progress leverages real-world clinical data of autistic individuals to ensure applicability for real-world situations. Demographic information is shown in Table 1, for both the patients and the pBTs. The patients had different ASD severities and number of prescribed ABA treatment hours; the pBTs were all females of different race/ethnicity and had varying types of health insurance coverage for their child.

**Table 1:** Descriptive data for the pBT-child dyads.

	pBT-child dyad 1	pBT-child dyad 2	pBT-child dyad 3
**Patients**			
**Age at Initial CFQL-2 (years)**	5.1	4.7	6.0
**Age at Follow- up CFQL-2 (years)**	5.7	5.4	6.9
**Sex Assigned at Birth**	Male	Female	Male
**Race, Ethnicity**	White, Hispanic or Latino	Black or African American, Not Hispanic or Latino	White, Hispanic or Latino
**ASD Severity**	Moderate	Severe	Moderate
**Prescribed Treatment Hours/Week**	30	40	25
**Comorbidities (Medications)**	none	chronic lung disease (albuterol, fluticasone, cetirizine)	none
**Insurance Type**	Public	Public	Private
**pBTs**			
**Sex Assigned at Birth**	Female	Female	Female
**Race, Ethnicity**	White, Hispanic or Latino	Black or African American, Not Hispanic or Latino	White, Not Hispanic or Latino

### Participants

**Fig 1:**
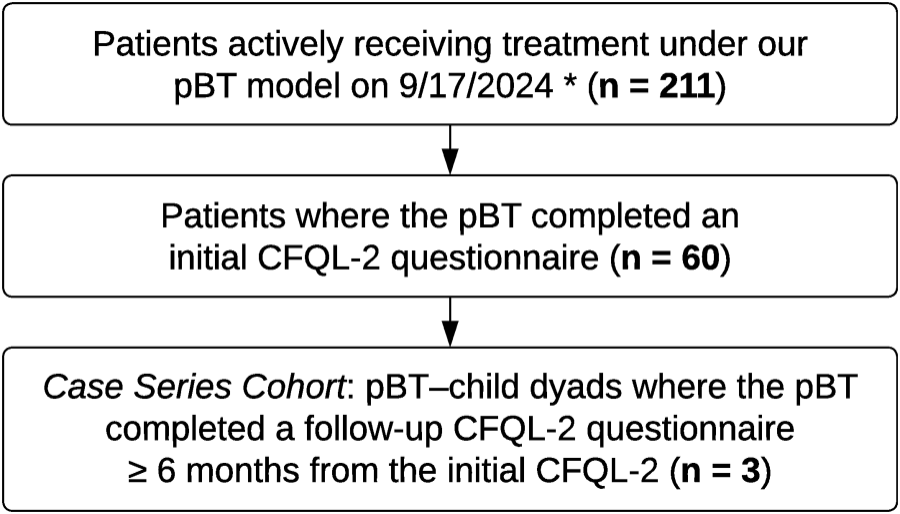
Attrition chart for selection and inclusion of dyads in the study cohort. * = the date when data was extracted. pBT = parent behavior technician; CFQL-2 = Child and Family Quality of Life-Second Edition.

pBTs with children (patients) who were actively receiving ABA treatment via our pBT model, and where the pBTs completed two CFQL-2 questionnaires a minimum of six months apart by September 2024 (when data were extracted) were eligible for inclusion. The attrition of the dyads was based on whether the pBTs completed the CFQL-2 questionnaires. From the 211 dyads with patients actively receiving ABA treatment on 9/17/2024, only 60 pBTs completed the first CFQL-2 questionnaire; and from these 60 dyads, only three pBTs completed the second CFQL-2 questionnaire. Thus, a total of three families had the necessary data for inclusion, as shown in the attrition chart (Fig 1).

### ABA treatment delivery

The three pBT-child dyads investigated in this case series all initiated ABA treatment using the pBT treatment model. This model relies on a data-driven, technology-facilitated approach to allow families to access ABA treatment using telehealth tools. Enrolled parents received 50+ hours of synchronous and asynchronous instruction using a remote training platform in compliance with the Behavior Analyst Certification Board (BACB) SOC for ABA methods. After completing training, parents passed an Initial Competency Assessment to become qualified as BTs. Once this training phase was completed, parents worked closely with board-certified behavior analysts (BCBAs) to implement ABA treatment in alignment with the SOC. In addition to providing treatment oversight, BCBAs developed individualized treatment plans for each patient, which included prescribing a set number of treatment hours per week for each patient while taking into consideration their specific needs (e.g., medical history, behavioral assessments, etc.) and identifying the IBs to target. pBTs delivered ABA to their child using detailed treatment directions from the BCBA. The overall aim of the treatment was to reduce IBs and encourage uptake of constructive behaviors, by acquiring skills in several focus areas (FAs) (communication/COM; emotional regulation/ER; executive functioning/EF; social skills/SS).

### Outcome measures

The CFQL-2 was adapted from the CFQL, First Edition (CFQL-1) to guide clinical intervention with regard to the impact ASD and child developmental disorders have on family QoL, with the added features of having fewer questions than CFQL-1 to minimize burden and provide greater sensitivity when evaluating QoL changes over time [15]. The CFQL-2 allows clinicians to track change over time across multiple QoL domains, including between administrations of the test for the same individual [29]. A study concluded that test-retest reliability of the CFQL-2 was significant, per a Pearson correlation coefficients test [29,30]. The 26-item CFQL-2 assessment is performed using a 5-point Likert scale, and is designed to assess seven psychosocial QoL domains: child, family, caregiver, partner relationship (PR), social network (SN), coping, and financial [16]. Only the parent from the third dyad provided responses for the relationship domain. CFQL-2 responses are intended to capture QoL among the included domains for up to one month prior to when the individual completes the assessment [29]. Parent overall QoL is reflected as the total score. Parents completed an initial CFQL-2 before initiating treatment with their child, then took a follow-up CFQL-2 at least 6 months later (range: 7–9 months after initial CFQL-2). Calculation of scores from the Likert scale to determine level of QoL on a categorical scale of low through high is described by Frazier and scoring is automated in the questionnaire platform [29].

pBTs tracked treatment progress in the pBT-facing interface of our proprietary application (“app”), designed to facilitate streamlined monitoring of patient clinical progress, by documenting specific progress towards targeted skill acquisition goals (SAGs), the incidence of IBs, as well as the number of treatment hours delivered. To track SAGs, the weekly average of successful attempts to perform a targeted skill (reported as percentage of attempts successfully completed) were tracked over the course of patient treatment. Similarly, the weekly total occurrence number for each IB was tracked over the course of treatment. These metrics assist the BCBAs in monitoring the progress of each patient in the context of their individual treatment plans, but also serve as detailed outcome measures which can be associated with other outcomes of interest, such as family QoL.

### Community involvement statement

The stakeholders within the ASD community that were involved in this research included parents of patients receiving ABA, with the parents providing primary treatment for their child, and the patients receiving ABA. Involvement of these stakeholders allowed us to leverage real-world data. Stakeholders were not involved in the study design, as data were collected observationally during the course of treatment, and used retrospectively for analysis. Therefore, no modifications were made to treatment for the purpose of this study.

### Ethics statement

All data were de-identified before analysis to ensure compliance with HIPAA and in accordance with the 1964 Helsinki Declaration, as revised in 2013. This study protocol was reviewed and approved by an independent Institutional Review Board on 10/20/2022 and received an exemption determination per 21CFR56.104 and 45CFR46.104(b)(4). Informed consent was obtained from adult study participants, for both their participation as well as their minor child’s participation.

## Case descriptions and analysis

### Parent-child dyad 1

**Fig 2:**
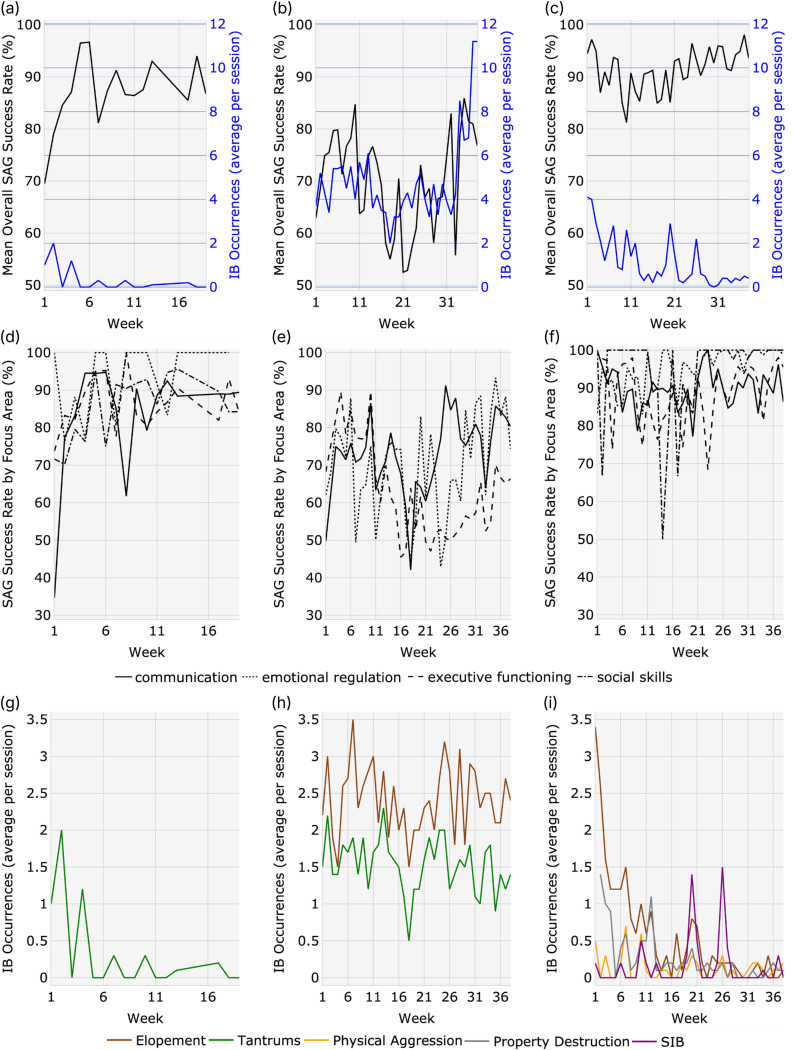
SAG success rates and IB occurrences. **(a)**, **(b)**, **(c)** – overall weekly SAG success rates and IB occurrences/session for the patient in dyad 1, 2, 3, respectively; **(d)**, **(e)**, **(f)** – weekly SAG success rate for each FA for the patient in dyad 1, 2, 3, respectively; and **(g)**, **(h)**, **(i)** – IB occurrences/session for each IB targeted for the patient in dyad 1, 2, 3, respectively. SAG = skill acquisition goal; IB = interfering behavior.

The first parent-child dyad consisted of a female pBT and her 5.1 year-old, male child diagnosed with non-syndromic [31] moderate ASD. The family was covered by public health insurance, which may be associated with relatively lower socioeconomic status (SES) [32,33]. Table 2 displays the CFQL-2 domain measures, with complete CFQL-2 data available in the S1 Table in S1 File, for all dyads. Treatment was prescribed by the BCBA at 30 hours/week, and the patient had a mean utilization of prescribed treatment of 49% (i.e., an average of 14.7 treatment hours/week). In this dyad, three QoL domains displayed a reliable change between the initial and the follow-up CFQL-2. Both the financial and SN domains improved substantially from the predefined categories of very low to average QoL. The caregiver domain was the only domain to reliably worsen, although the QoL category remained average. Regarding treatment progress (data available in the S2 Table in S1 File), the patient demonstrated an increase in the mean overall SAG success rate from 70% to 87% over 19 weeks (Fig 2(a)), with three FAs (COM, EF, SS) showing increases and one FA (ER) showing a decrease in the SAG success rate (Fig 2(d)). The greatest gains were observed in COM. IB reduction targeting the patient’s tantrums demonstrated substantial extinction (Fig 2(g)). The functions of the patient’s IB were identified by the BCBA in the treatment plan as avoidance, escape, and seeking attention; to replace this IB, the patient worked on the skills of manding to others and expressing internal state.

**Table 2:** CFQL-2 descriptive statistics.

	Initial Standard Score	Initial QoL Description	Follow-up Standard Score	Follow-up QoL Description	Standard Score Difference	Reliable Change^*^
**pBT-child dyad 1**						
**Total**	77.2	Low	89.4	Average	12.2	–
**Child**	88.8	Average	89.2	Average	0.4	–
**Family**	79.5	Low	90.2	Average	10.7	–
**Caregiver**	114.8	Average	97.7	Average	−17.1	Worsened
**Financial**	67.0	Very Low	106.0	Average	39.0	Improved
**SN**	62.7	Very Low	88.5	Average	25.8	Improved
**Coping**	91.8	Average	100.7	Average	8.9	–
**CFQL-2 CS**	93.8	Stable	100.0	Stable	6.2	–
**pBT-child dyad 2**						
**Total**	77.5	Low	82.0	Low	4.5	–
**Child**	72.3	Low	80.7	Low	8.4	–
**Family**	68.3	Very Low	62.7	Very Low	−5.6	–
**Caregiver**	103.0	Average	115.3	High	12.3	–
**Financial**	76.3	Low	56.4	Very Low	−19.9	Worsened
**SN**	89.5	Average	108.8	Average	19.3	Improved
**Coping**	101.2	Average	101.2	Average	0.0	–
**CFQL-2 CS**	101.0	Stable	89.4	Stable	−11.6	–
**pBT-child dyad 3**						
**Total**	67.9	Very Low	70.3	Low	2.4	–
**Child**	97.3	Average	89.9	Average	−7.4	–
**Family**	68.4	Very Low	62.8	Very Low	−5.6	–
**Caregiver**	74.6	Low	92.7	Average	18.1	Improved
**Financial**	76.5	Low	66.4	Very Low	−10.1	–
**SN**	62.6	Very Low	69.0	Very Low	6.4	–
**PR**	81.5	Low	91.1	Average	9.6	–
**Coping**	91.8	Average	82.8	Low	−9.0	–
**CFQL-2 CS**	89.0	Stable	94.3	Stable	5.3	–

CFQL-2 = Child and Family Quality of Life-Second Edition; SN = social network; CS = change scale; PR = partner relationship.

* An indication of “Reliable Change from Previous Assessment” is estimated and provided by the CFQL-2 assessment platform [29].

### Parent-child dyad 2

The second parent-child dyad consisted of a female pBT and her 4.7-year-old, female child diagnosed with non-syndromic [31] severe ASD. This family was also covered by public health insurance. Treatment was prescribed by the BCBA at 40 hours/week, and the patient had a mean utilization of prescribed treatment of 77% (i.e., average of 30.8 treatment hours/week). In this dyad, two QoL domains displayed a reliable change between the initial and the follow-up CFQL-2. The SN improved substantially, although it categorically remained average. The financial domain worsened, and QoL went from low to very low. The total QoL, as well as child, family, caregiver, and coping domains, and the overall change score, were stable. Regarding treatment progress, the patient demonstrated an increase in the mean overall SAG success rate from 52% to 86% over 38 weeks (Fig 2(b)), with two FAs showing increases (COM, ER) and one FA (EF) showing an overall decrease (Fig 2(e)). The greatest gains were observed in COM. IB reduction targeting the patient’s elopement and tantrums demonstrated, respectively, no substantial change and a slight decrease in the number of occurrences (Fig 2(h)). The functions of the patient’s IBs were identified by the BCBA in the treatment plan as escape and avoidance; to replace these IBs, the patient worked on the skills of manding to others, generalizing mands, responding to others, labeling, successful transitions, assent, interactive play, responding to basic safety commands, and complex behavior chain including multiple steps.

### Parent-child dyad 3

The third parent-child dyad consisted of a female pBT and her 6.1-year-old, male child diagnosed with non-syndromic [31] moderate ASD. The family was covered by private health insurance, which may be associated with a relatively higher SES [34]. Treatment was prescribed by the BCBA at 25 hours/week and the patient had a mean utilization of 128% (i.e., average of 32.1 treatment hours/week). In this dyad, one QoL domain displayed a reliable change, with substantial improvement in the caregiver domain, which increased from low to average. Regarding clinical progress, the patient demonstrated a stable mean overall SAG success rate of 95% over 38 weeks (Fig 2(f)), with three FAs (COM, ER, SS) showing an increase and one FA (EF) showing a decrease in SAG success rate (Fig 2(g)). The greatest gains were observed in ER. IB reduction targeting the patient’s elopement and property destruction demonstrated substantial extinction; physical aggression and self-injurious behaviors remained approximately stable (Fig 2(h)). The functions of the patient’s IBs were identified by the BCBA in the treatment plan as escape, avoidance, and seeking attention; to replace these IBs, the patient worked on the skills of successful transitions, collaborative play, manding to others, manding for information, attending/activity engagement, utilizing token economy system, and etiquette.

## Discussion

This study explored QoL outcomes for three parent-child dyads utilizing the pBT model for delivery of ABA, as well as examining how patient outcomes in terms of clinical progress correlate with QoL change using the CFQL-2 total and subscales. It was previously shown that across the CFQL-2 total and subscales used to quantify QoL, there is adequate to excellent reliability in the ranges of the latent trait important for discriminating impaired QoL from average QoL; with a lowered reliability when distinguishing average QoL from high or very high QoL [29,30]. In terms of QoL improvement for the pBTs, overall, the results of this exploratory investigation indicated that all three pBTs achieved overall improvement in QoL between the first and follow-up evaluation, as indicated by CFQL-2 scores. However, the clinical significance of this change may be small in some cases, as indicated by the small number of domains which showed reliable score increases for each dyad. These included improvement in the financial and SN domains for dyad one, SN for dyad two, and caregiver for dyad three.

The largest increase in overall CFQL-2 score was seen in parent-child dyad 1, driven primarily by the financial and SN domains. Previous investigations of parental stress in the context of ASD have found that both the overall severity of ASD diagnosis, as well as specific IBs, are associated with lower parental QoL. While this patient presented with ASD of moderate severity, they only had one IB targeted for reduction in their treatment plan (tantrums), which showed marked extinction and was virtually eliminated after only 19 weeks of treatment. It seems intuitive to speculate that the reduction in tantrums and the fact that this patient had only one IB to address contributed to improved QoL in this dyad, although this change does not obviously translate to the domains which were most improved. Some reports indicate that parents of children on the autism spectrum avoid social environments due to the negative interactions they experience when IBs occur in these settings. This parent may have been able to more effectively seek social support after their child’s tantrums improved; alternately or additionally, the resources and support provided by the pBT model may have served to improve their social network. SAG success rate also increased for this patient; an increase in constructive skills related to COM, EF, and SS may also contribute to improving QoL. The only QoL area which reliably decreased was the caregiver domain. This was not observed in the other parent-child dyads investigated, however, it is possible that the increased parental involvement promoted by the pBT model represented an additional responsibility which added to the overall caregiving stress experienced by this parent.

Similarly to the first dyad, parent-child dyad 2 showed a reliable increase in the SN domain of the CFQL-2, although the overall score increase was smaller in magnitude. The child in this dyad presented with severe ASD symptoms, and, unlike the first dyad, did not show the same magnitude of IB reduction over the course of treatment for either elopement or tantrums. These patient factors would suggest a higher level of parental stress contributing to decreased QoL in this dyad, which is reflected in the low categorical rankings in QoL domains for both CFQL-2 assessments. It is remarkable to see the increase in SN given these poor prognostic indicators for QoL, though a struggle in achieving other QoL improvements may be attributed to the severity of patient’s symptoms, which contributes to a higher level of parental stress [35], linked with reduced QoL [10]. Given the persistent IBs for this child, resources provided by the pBT model, such as logistical support, treatment plan structure, clinician oversight, and technological tools, and on-demand training resources and support, may have played a significant role in improving the SN support experienced by this dyad. The improvement this child showed in the SAG FAs of COM and ER should also be considered for their potential contributions to improved QoL. The only CFQL-2 area which showed a reliable decrease for this dyad was the financial domain, which declined from low to very low. The parental time commitment involved for ABA delivery, especially for a patient prescribed a comprehensive, 40 hour/week treatment plan, is substantial and may have contributed to the financial strain experienced by this dyad.

Parent-child dyad 3 showed the smallest magnitude of overall CFQL-2 increase of the 3 dyads in this case series, but was also the only dyad which did not experience a reliable decrease in any specific QoL domain. This was also the only of the three dyads to show a reliable increase in the caregiver domain, which changed from a ranking of low to average. This parent-child dyad exhibited both a very high rate of treatment utilization (>100%) and high SAG success rate over the course of treatment (95%), which exceeded the 80% threshold that is used to determine skill mastery. COM was the only SAG FA which showed a small decline over the treatment period, with the other three FAs showing slight improvements; however, in line with the 80% mastery criteria, this decline did not demonstrate a loss of skills [36]. There were four different IBs targeted for reduction, two of which were substantially eliminated (elopement and property destruction) and two of which continued to occur sporadically throughout the treatment period (physical aggression and self-injurious behaviors). Given the potential severity of consequences associated with all four of these IBs, even small decreases in their incidence or magnitude are likely to be impactful to family stress and QoL. The large improvement in caregiver QoL for this dyad may be related to an increase in parental self-efficacy associated with this parent gaining the tools and resources needed to make meaningful progress in reducing their child’s IBs. Additionally, this improvement in caregiver QoL may also be associated with the parent being the only White (Not Hispanic or Latino) pBT in the study, and/or with this being the only family with private health insurance, both of which indicate the potential for a higher SES.

### Study limitations

The case series study design poses several limitations. The selection of these three parent-child dyads represented an opportunity sample, because this was not a study specifically designed around participant retention, and parent attrition may have resulted in biased sample selection. Further, the differences in patient age, ASD severity, and number of hours of prescribed ABA treatment may account for some of the differences in the patient’s response to treatment in terms of acquiring skills as well as reducing IBs. Data presented herein primarily relies on results self-reported by parents, which may be subject to confirmation bias as parents anticipate improvement in their child’s behavior over time. Furthermore, while we posit a connection between parental self-efficacy and the outcome variables of interest (stress, QoL), it was out of the scope of this study to examine parental self-efficacy.

### Future directions

This preliminary investigation on the relationship between QoL and clinical progress in the pBT model of ABA delivery indicates that treatment progress, especially with regard to reducing IBs, may play an important role in improving QoL for families with children on the autism spectrum. Although not the focus of this investigation, we speculate that this relationship is mediated by the combined effects of reducing stress associated with the consequences of IBs and by increasing parental self-efficacy through the provision of knowledge and tools for effective ABA delivery. This speculation is supported by ample evidence linking self-efficacy and parental stress in the context of ASD [37].

Future investigations will utilize larger parent-child dyad cohorts in order to assess the population-level significance of changes within CFQL-2 domains over the course of ABA treatment. Further, we plan to incorporate direct assessments of parental self-efficacy, such as self-reported data collected via the Family Empowerment Scale [38], to gain a more robust understanding of the relationship between self-efficacy, stress, and family QoL. Lastly, future work may involve identifying mechanisms to address self-efficacy, stress, and QoL to support development of highly personalized treatment plans, and the impact that these tailored interventions have on family and patient outcomes.

## Conclusions

This case series investigation reports on the change in QoL, as assessed by CFQL-2, in three parent-child dyads receiving treatment through the pBT model of ABA delivery. All three dyads showed increases in overall CFQL-2 score, though the magnitude and reliability of changes varied between the dyads with regard to specific QoL domains. Similarly, all three dyads showed clinical progress in the reduction of targeted IBs and success towards SAGs, but with varied progress seen with regard to specific IBs and SAG FAs. These preliminary findings highlight the heterogeneity of families with children on the autism spectrum, and the need for comprehensive investigation of the complex relationships between individual factors, family stress and QoL, and ABA clinical outcomes.

## Supporting information

S1 File **S1 Table**. CFQL-2 descriptive statistics. QoL = quality of life; Avg = average; CFQL-2 = Child and Family Quality of Life-Second Edition; SN = social network; CS = change scale; PR = partner relationship. **S2 Table**. Skill acquisition goals and interfering behavior data.
(ZIP)
