# Supplementary material for: Quality of life in a family-centric applied behavior analysis model: A case series study
Source: PLoS One. 2025 Aug 6;20(8):e0329939. doi: 10.1371/journal.pone.0329939 (PMC12327611; doi:10.1371/journal.pone.0329939)
Supplement: S1 File — S1 Table. CFQL-2 descriptive statistics. QoL = quality of life; Avg = average; CFQL-2 = Child and Family Quality of Life-Second Edition; SN = social network; CS = change scale; PR = partner relationship. S2 Table. Skill acquisition goals and interfering behavior data. (ZIP) [file pone.0329939.s001.zip › S1_Table.docx]

**Supporting information**

**S1 Table. CFQL-2 Descriptive Statistics.** QoL = quality of life; Avg = average; CFQL-2 = Child and Family Quality of Life-Second Edition; SN = social network; CS = change scale; PR = partner relationship.

|  | **Initial Standard Score** | **Initial Percentile** | **Initial QoL Description** | **Follow-up Standard Score** | **Follow-up Percentile** | **Follow-up QoL Description** | **Standard Score Difference** | **Reliable Change^*^** |
| --- | --- | --- | --- | --- | --- | --- | --- | --- |
| **pBT-child dyad 1** | | | | | | | | |
| **Total** | 77.2 | 6.4 | Low | 89.4 | 24.0 | Avg | 12.2 | - |
| **Child** | 88.8 | 22.8 | Avg | 89.2 | 23.6 | Avg | 0.4 | - |
| **Family** | 79.5 | 8.5 | Low | 90.2 | 25.7 | Avg | 10.7 | - |
| **Caregiver** | 114.8 | 83.8 | Avg | 97.7 | 43.9 | Avg | -17.1 | Worsened |
| **Financial** | 67.0 | 1.4 | Very Low | 106.0 | 65.6 | Avg | 39.0 | Improved |
| **SN** | 62.7 | 0.6 | Very Low | 88.5 | 22.2 | Avg | 25.8 | Improved |
| **Coping** | 91.8 | 29.2 | Avg | 100.7 | 51.9 | Avg | 8.9 | - |
| **CFQL-2 CS** | 93.8 | 34.1 | Stable | 100.0 | 49.9 | Stable | 6.2 | - |
| **pBT-child dyad 2** | | | | | | | | |
| **Total** | 77.5 | 6.7 | Low | 82 | 11.5 | Low | 4.5 | - |
| **Child** | 72.3 | 3.2 | Low | 80.7 | 10.0 | Low | 8.4 | - |
| **Family** | 68.3 | 1.7 | Very Low | 62.7 | 0.6 | Very Low | -5.6 | - |
| **Caregiver** | 103.0 | 57.9 | Avg | 115.3 | 84.6 | High | 12.3 | - |
| **Financial** | 76.3 | 5.7 | Low | 56.4 | 0.2 | Very Low | -19.9 | Worsened |
| **SN** | 89.5 | 24.2 | Avg | 108.8 | 72.1 | Avg | 19.3 | Improved |
| **Coping** | 101.2 | 53.1 | Avg | 101.2 | 53.1 | Average | 0.0 | - |
| **CFQL-2 CS** | 101.0 | 52.8 | Stable | 89.4 | 23.9 | Stable | -11.6 | - |
| **pBT-child dyad 3** | | | | | | | | |
| **Total** | 67.9 | 1.6 | Very Low | 70.3 | 2.4 | Low | 2.4 | - |
| **Child** | 97.3 | 42.8 | Avg | 89.9 | 25.0 | Avg | -7.4 | - |
| **Family** | 68.4 | 1.8 | Very Low | 62.8 | 0.7 | Very Low | -5.6 | - |
| **Caregiver** | 74.6 | 4.5 | Low | 92.7 | 31.4 | Avg | 18.1 | Improved |
| **Financial** | 76.5 | 5.8 | Low | 66.4 | 1.2 | Very Low | -10.1 | - |
| **SN** | 62.6 | 0.6 | Very Low | 69.0 | 1.9 | Very Low | 6.4 | - |
| **PN** | 81.5 | 10.9 | Low QoL | 91.1 | 27.7 | Average QoL | 9.6 | - |
| **Coping** | 91.8 | 29.2 | Avg | 82.8 | 12.6 | Low | -9.0 | - |
| **CFQL-2 CS** | 89.0 | 23.2 | Stable | 94.3 | 35.3 | Stable | 5.3 | - |

* An indication of “Reliable Change from Previous Assessment” is estimated and provided by the CFQL-2 assessment platform [(Frazier, 2020)](https://web.endnote.com/citations/eyJkaXNwbGF5VGV4dCI6IihGcmF6aWVyLCAyMDIwKSIsImNpdGF0aW9ucyI6W3sicmVjb3JkIjp7InRpdGxlcyI6eyJ0aXRsZSI6IkNoaWxkIGFuZCBGYW1pbHkgUXVhbGl0eSBvZiBMaWZlLCAoQ0ZRTC0yKSBNYW51YWwifSwidXJscyI6eyJyZWxhdGVkLXVybHMiOnsidXJsIjoiaHR0cHM6Ly9hdXRpc21hbmFseXRpY2EtcHVibGljLWFzc2V0cy5zMy5hbWF6b25hd3MuY29tL1BST0QtMTM2MytDaGlsZCthbmQrRmFtaWx5K1F1YWxpdHkrb2YrTGlmZSsoQ0ZRTCkrTWFudWFsKygxKS5wZGYifX0sInB1Ymxpc2hlciI6IkF1dGlzbSBBbmFseXRpY2EiLCJyZWYtdHlwZSI6IjE3IiwicmVjLXVzbiI6IjEzMTgiLCJjb250cmlidXRvcnMiOnsiYXV0aG9ycyI6eyJhdXRob3IiOiJGcmF6aWVyLCBUaG9tYXMgVyJ9fSwibnVtYmVyIjoiMjAyMCIsInJlYy1ndWlkIjoiZDg5MWEwNjctZDI2Ny00ZjI3LTk4M2UtZDlhMWEzZDI4OWRjIiwiZGF0ZXMiOnsieWVhciI6IjIwMjAiLCJwdWItZGF0ZXMiOnsiZGF0ZSI6IjIwMjAifX19LCJndWlkIjoiZDg5MWEwNjctZDI2Ny00ZjI3LTk4M2UtZDlhMWEzZDI4OWRjIiwiYmlibGlvQ29udGVudCI6W3siZGF0ZSI6IjIwMjAiLCJncm91cEd1aWRzIjpbXSwidXJsIjpbImh0dHBzOi8vYXV0aXNtYW5hbHl0aWNhLXB1YmxpYy1hc3NldHMuczMuYW1hem9uYXdzLmNvbS9QUk9ELTEzNjMrQ2hpbGQrYW5kK0ZhbWlseStRdWFsaXR5K29mK0xpZmUrKENGUUwpK01hbnVhbCsoMSkucGRmIl0sInllYXIiOiIyMDIwIiwidGl0bGUiOiJDaGlsZCBhbmQgRmFtaWx5IFF1YWxpdHkgb2YgTGlmZSwgKENGUUwtMikgTWFudWFsIiwicHVibGlzaGVyIjoiQXV0aXNtIEFuYWx5dGljYSIsInJlZmVyZW5jZVR5cGUiOiIxNyIsInJzeG1sIjoiPHJlY29yZD48cmVmLXR5cGU%2BMTc8L3JlZi10eXBlPjxjb250cmlidXRvcnM%2BPGF1dGhvcnM%2BPGF1dGhvcj5GcmF6aWVyLCBUaG9tYXMgVzwvYXV0aG9yPjwvYXV0aG9ycz48L2NvbnRyaWJ1dG9ycz48dGl0bGVzPjx0aXRsZT5DaGlsZCBhbmQgRmFtaWx5IFF1YWxpdHkgb2YgTGlmZSwgKENGUUwtMikgTWFudWFsPC90aXRsZT48L3RpdGxlcz48ZGF0ZXM%2BPHllYXI%2BMjAyMDwveWVhcj48cHViLWRhdGVzPjxkYXRlPjIwMjA8L2RhdGU%2BPC9wdWItZGF0ZXM%2BPC9kYXRlcz48cHVibGlzaGVyPkF1dGlzbSBBbmFseXRpY2E8L3B1Ymxpc2hlcj48dXJscz48cmVsYXRlZC11cmxzPjx1cmw%2BaHR0cHM6Ly9hdXRpc21hbmFseXRpY2EtcHVibGljLWFzc2V0cy5zMy5hbWF6b25hd3MuY29tL1BST0QtMTM2MytDaGlsZCthbmQrRmFtaWx5K1F1YWxpdHkrb2YrTGlmZSsoQ0ZRTCkrTWFudWFsKygxKS5wZGY8L3VybD48L3JlbGF0ZWQtdXJscz48L3VybHM%2BPG51bWJlcj4yMDIwPC9udW1iZXI%2BPHJlYy1ndWlkPmQ4OTFhMDY3LWQyNjctNGYyNy05ODNlLWQ5YTFhM2QyODlkYzwvcmVjLWd1aWQ%2BPHJlYy11c24%2BMTMxODwvcmVjLXVzbj48L3JlY29yZD4iLCJyZWNvcmRTdGF0dXMiOiJhY3RpdmUiLCJhdXRob3JzIjpbIkZyYXppZXIsIFRob21hcyBXIl0sImd1aWQiOiJkODkxYTA2Ny1kMjY3LTRmMjctOTgzZS1kOWExYTNkMjg5ZGMiLCJudW1iZXIiOiIyMDIwIn1dfV19).

[Frazier, T. W. (2020). Child and Family Quality of Life, (CFQL-2) Manual. (2020).](https://web.endnote.com/reference-list/)
